# RNA-seq analysis of galaninergic neurons from ventrolateral preoptic nucleus identifies expression changes between sleep and wake

**DOI:** 10.1186/s12864-020-07050-7

**Published:** 2020-09-14

**Authors:** Xiaofeng Guo, Xiaoling Gao, Brendan T. Keenan, Jingxu Zhu, Dimitra Sarantopoulou, Jie Lian, Raymond J. Galante, Gregory R. Grant, Allan I. Pack

**Affiliations:** 1 Division of Sleep Medicine/Department of Medicine, University of Pennsylvania Perelman School of Medicine, Philadelphia, 19104 USA; 2 Department of Respiratory and Critical Care Medicine, Second Hospital of Shanxi Medical University, Taiyuan, 030001 Shanxi China; 3 Institute for Translational Medicine and Therapeutics, University of Pennsylvania, Philadelphia, 19104 USA; 4 Present address at National Institute on Aging, National Institutes of Health, Baltimore, 21224 USA; 5 Department of Genetics, University of Pennsylvania, Philadelphia, 19104 USA

**Keywords:** Ventrolateral preoptic nucleus, Galaninergic neurons, Spontaneous sleep, Sleep deprivation, Next-generation RNA-sequencing

## Abstract

**Background:**

Previous studies show that galanin neurons in ventrolateral preoptic nucleus (VLPO-Gal) are essential for sleep regulation. Here, we explored the transcriptional regulation of the VLPO-Gal neurons in sleep by comparing their transcriptional responses between sleeping mice and those kept awake, sacrificed at the same diurnal time.

**Results:**

RNA-sequencing (RNA-seq) analysis was performed on eGFP(+) galanin neurons isolated using laser captured microdissection (LCM) from VLPO. Expression of *Gal* was assessed in our LCM eGFP(+) neurons via real time qPCR and showed marked enrichment when compared to LCM eGFP(−) cells and to bulk VLPO samples. Gene set enrichment analysis utilizing data from a recent single-cell RNA-seq study of the preoptic area demonstrated that our VLPO-Gal samples were highly enriched with galanin-expressing inhibitory neurons, but not galanin-expressing excitatory neurons. A total of 263 genes were differentially expressed between sleep and wake in VLPO-Gal neurons. When comparing differentially expressed genes in VLPO-Gal neurons to differentially expressed genes in a wake-active neuronal region (the medial prefrontal cortex), evidence indicates that both systemic and cell-specific mechanisms contribute to the transcriptional regulation in VLPO-Gal neurons. In both wake-active and sleep-active neurons, ER stress pathways are activated by wake and cold-inducible RNA-binding proteins are activated by sleep. In contrast, expression of DNA repair genes is increased in VLPO-Gal during wakefulness, but increased in wake-active cells during sleep.

**Conclusion:**

Our study identified transcriptomic responses of the galanin neurons in the ventrolateral preoptic nucleus during sleep and sleep deprivation. Data indicate that VLPO contains mainly sleep-active inhibitory galaninergic neurons. The VLPO galanin neurons show responses to sleep and wake similar to wake-active regions, indicating these responses, such as ER stress and cold-inducible RNA-binding proteins, are systemic affecting all neuronal populations. Region-specific differences in sleep/wake responses were also identified, in particular DNA repair. Our study expands knowledge about the transcriptional response of a distinct group of neurons essential for sleep.

## Background

The preoptic area (POA) of the hypothalamus, particularly the ventrolateral preoptic area (VLPO), is essential for sleep regulation [46]. Damage to POA causes insomnia-like sleep disturbances in rats and cats, and the severity of sleep disturbances are correlated to the degree of damage [23, 37, 54]. Single neuron recordings in POA, including VLPO, demonstrated elevated discharge during non-rapid eye movement (NREM) and rapid eye movement (REM) sleep compared to waking, and the degree of increase reflected sleep depth, indicating their possible involvement in sleep homeostasis [27, 52, 53]. Studies using c-Fos immunoreactivity to indicate recent neuronal activities demonstrated increased numbers of c-Fos positive neurons in VLPO following consolidated sleep compared to wake, and the number of c-Fos positive neurons increased with recovery sleep after sleep deprivation, and showed a positive correlation with the amount of sleep before sacrifice [17, 18, 50]. These sleep-active neurons, particularly from VLPO, project to histaminergic tuberomammillary nucleus (TMN), serotonergic dorsal raphe (DR), and noradrenergic locus coreleus (LC), and the majority are GABAergic inhibitory neurons, suggesting that during sleep they inhibit multiple monoamine arousal systems [49, 51].

Studies show that approximately 80% of the neurons that project to TMN from VLPO express galanin [49], and this is conserved across multiple mammalian species [16]. In a recent study, optogenetic and chemogenetic tools were applied to specifically activate or inhibit VLPO galanin neurons (VLPO-Gal), demonstrating that VLPO-Gal neurons were sleep-active and sleep-promoting [28]. Chung et al. had conflicting results regarding the function of VLPO-Gal neurons [9]; however, this is likely related to the optogenetic stimulation being at too high a frequency, resulting in depolarization block [28]. In the present study, we aimed to further characterize the galaninergic neurons in VLPO by examining their behavioral state dependent transcriptional regulation between sleep and wake.

Galanin neuron localization using immunohistochemistry is challenging because galanin peptide is not mainly located in the cell body, and requires the use of colchicine to block axonal transport. However, this induces cellular stress [29, 60]. Hence, we utilized Tg (Gal-EGFP)HX109Gsat mice that express eGFP (enhanced green fluorescent protein) under control of the galanin promoter to aid identification of galanin-expressing neurons in VLPO. Laser capturing microdissection (LCM) was used to isolate individual eGFP-expressing cells. We used our previous design [34], comparing gene expression at 3, 6, 9, and 12 h after lights-on (7 AM) between sleeping mice and those kept awake by gentle handling [14]. The same study design has been used in multiple published transcriptomics studies, including surveying bulk tissues of multiple brain regions [cortex and hypothalamus [34]] and peripheral tissues [lung and heart [2]], as well as LCM isolated cholinergic neurons in basal forebrain using microarrays [42]. The same design was also used in a recent bulk tissue study of medial prefrontal cortex using RNA-seq [20]. The similarity of the approach used here and in these prior studies allowed us to compare genes differentially expressed between sleep and wake in VLPO-Gal neurons to genes found differentially regulated in different regions and cell-types.

## Results

### LCM enriched galanin expressing neurons from VLPO

**Fig. 1:**
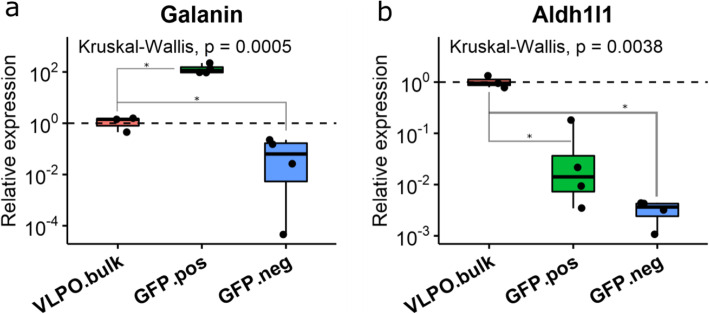
Confirmation for the enrichment of galanin-expressing neurons in eGFP(+) samples collected using LCM. **a** eGFP(+) samples showed a median 116.9-fold increase in the expression of Gal when compared to the VLPO bulk tissues, indicating effective enrichment of galanin-expressing neurons in our eGFP(+) LCM samples. **b** Expression of the astrocyte gene *Aldh1l1* in both the eGFP(+) and eGFP(−) samples was significantly lower when compared to the VLPO bulk tissues, indicating effective removal of contaminating astrocytes from the neuron samples collected by LCM. Kruskal-Wallis tests were made for comparisons among all three groups. Separate pairwise comparisons were then made between the VLPO bulk tissues and the LCM samples (eGFP+ and eGFP-) using one-tailed Wilcoxon exact test (see Methods). Y-axis shows fold difference in gene expression relative to VLPO bulk tissue on log10 scale

Galanin neurons are located in a number of nuclei and sub-structures of VLPO in POA, in addition to the VLPO core. These include the dorsal and medial VLPO extensions, as well as the median, medial, and the periventricular preoptic nuclei, as described previously [28]. To preserve the RNA quality, a 3 mm brain slice containing the VLPO region was post-fixed with 4% formaldehyde on ice for 5-min. The short post-fix was sufficient for the GFP fluorescent detection in the VLPO core and the medial VLPO extension, but not in the dorsal extension of VLPO and beyond (Supplementary figure 1 in Additional file 1). eGFP(+) cells in VLPO core were dissected using LCM with care taken to avoid picking eGFP(+) cells in the medial VLPO extension, as shown in Supplementary figure 2 (Additional file 1). To validate enrichment of galanin expressing neurons in our LCM samples, we compared expression of *Gal* among the eGFP(+) neurons from the VLPO core, eGFP(−) cells from the adjacent piriform cortex on the same sections, and the bulk VLPO tissue (Bregma + 0.4 to − 0.1, lateral ±0.5 mm). Significant differences among the three sample types were observed for expression of both *Gal* (Kruskal-Wallis exact *p*-value = 0.0005) and *Aldh1l1* (*p* = 0.0038). As shown in Fig. 1a, expression of *Gal* in eGFP(+) samples showed a median fold-increase of 116.9 (95% confidence interval [CI]: 59.5, 497.0; *p* = 0.0286) when compared to VLPO bulk tissues, whereas the expression of *Gal* in eGFP(−) samples showed a median fold-reduction of 13.4 (95% CI: 2.0, 34,509.4; *p* = 0.0286) when compared to the VLPO bulk tissue. On the other hand, expression of *Aldh1l1*, a specific marker of astrocytes, was significantly decreased in both the eGPF(+) (median [95% CI] fold-decrease = 72.5 [4.4, 386.2]; *p* = 0.0286) and the eGFP(−) (median [95% CI] fold-decrease = 301.0 [179.4, 1252.9]; *p* = 0.0286) samples when compared to the VLPO bulk tissue (Fig. 1b). Thus, results indicate effective enrichment of galanin expressing neurons and removal of contaminating astrocytes in our VLPO-Gal samples using LCM.

### Differentially regulated genes in VLPO-gal neurons between sleep and sleep deprivation

**Fig. 2:**
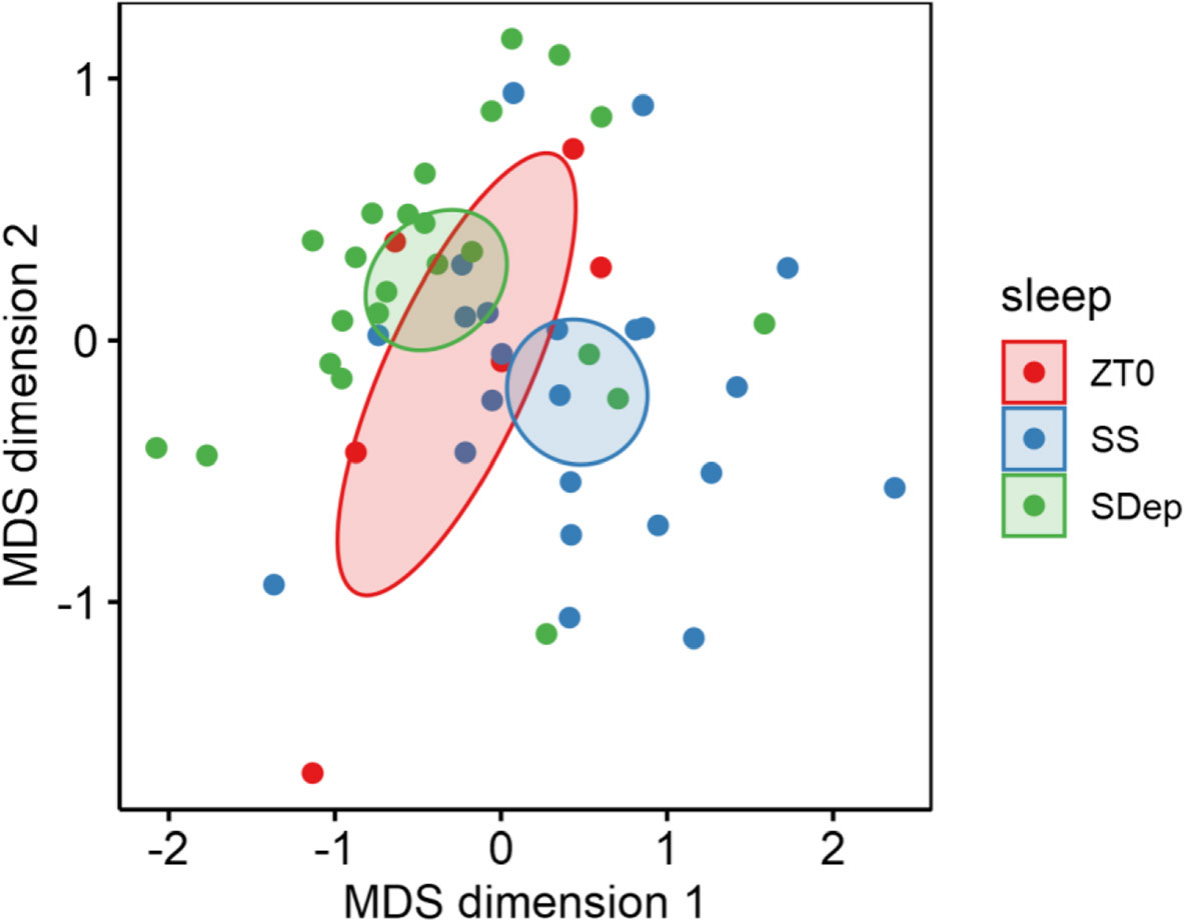
Plot of the multidimensional scaling (MDS) result. SS and SDep samples formed separate clusters separated primarily on the first dimension, demonstrating that behavioral state explains the largest proportion of gene expression variability. Mice collected at ZT0 were between the SS and SDep. Results are shown with confidence ellipse at 95% confidence intervals

We first examined the source of variation within the samples using unsupervised clustering of normalized gene expression data via multidimensional scaling (MDS) analysis. As shown in Fig. 2, samples from the spontaneous sleep mice (SS) were primarily separated from the samples from the sleep deprived mice (SDep) on the first dimension of the MDS plot, indicating behavioral state explains the greatest proportion of overall variability in the data. Samples collected at baseline (ZT0) fall between SS and SDep samples, which is consistent with the fact that this time point is prior to the onset of SS or SDep.

**Fig. 3:**
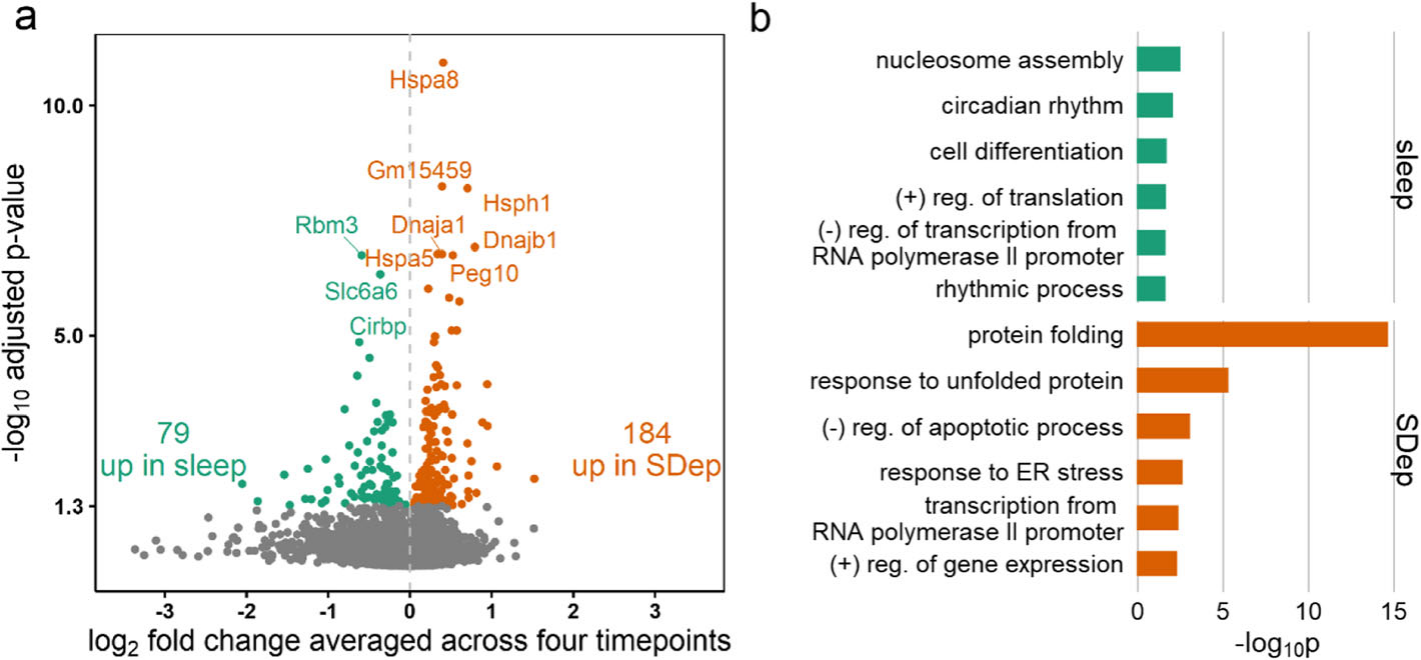
Differentially expressed genes between spontaneous sleep (SS) and sleep deprivation (SDep). **a** 184 genes were identified to be significantly up-regulated by SDep (orange) and 79 genes were identified to be significantly up-regulated by sleep (green) with a cutoff of FDR < 0.05. **b** Selected Biological Processes GO terms enriched in the differentially expressed genes. Protein folding, response to unfolded protein, and regulation of gene expression are among the functions enriched from the genes up-regulated in SDep (red), whereas nucleosome assembly and regulation of translation are among the functions enriched from the genes up-regulated in sleep (green). X-axis shows the negative log10 of the *P*-values of the GO enrichment

We next assessed differentially expressed genes (DEGs) in VLPO-Gal neurons between mice sleep deprived for 3, 6, 9 or 12 h starting from ZT0 and their time-matched SS controls using a moderated F-test in *LimmaVoom*. A total of 184 and 79 genes were up-regulated by SDep and SS, respectively, based on an FDR of 5% (Fig. 3a). The SDep up-regulated genes were strongly enriched for GO biological process (BP) terms related to protein folding, including response to unfolded protein and response to ER stress, whereas the genes up-regulated during SS were enriched for nucleosome assembly, circadian rhythm, and positive regulation of translation (Fig. 3b). In the molecular function (MF) and cellular component (CC) categories, among the genes up-regulated by SDep, unfolded protein binding, chaperone binding, and poly(A) RNA binding were the most significantly enriched MF terms, and nucleus and ER chaperone complex were the most significantly enriched CC terms. Among the genes up-regulated by SS, DNA-binding and nucleosome were significantly enriched MF and CC terms. These results agree with the results obtained using the BP category. The complete list of differentially expressed genes and DAVID gene ontology analysis results are provided in Additional file 2.

### Temporal changes in gene expression during sleep and sleep deprivation

We next incorporated the mice collected at ZT0 and evaluated the gene expression changes with time (from ZT0 to ZT12) during SS and SDep, separately. Among the 263 DEGs differentially regulated between SS and SDep, 7 genes were significantly associated with time during SS and 37 genes were significantly correlated with time during SDep at an FDR cutoff of 5% (Table 1). The three circadian-regulated transcription factors, *Dbp*, *Zbtb16*, and *Fkbp5*, changed with time in the same direction during both SS and SDep, suggesting that SDep affected the magnitude of the circadian gene expression, but not the direction of the circadian phase. For the rest of the genes, the direction of the associations matched the behavior state-regulated gene expression changes. For example, *Rbm3* was down-regulated by SDep when compared to the time-matched SS controls, and its expression associated positively with time during SS and negatively with time during SDep (Table 1).

**Table 1:** Correlation with time during SS or SDep among genes differentially regulated between SS and SDep

**Correlation with time during spontaneous sleep (SS)**		
Genes	Correlation with time	Affected by SS (vs. SDep)
Dbp, Gpcpd1, Rbm3	positive	**↑ up-regulated with SS**
Zbtb16, Fkbp5	positive	**↓ down-regulated with SS**
Stip1, Cct7	negative	**↓ down-regulated with SS**
**Correlation with time during sleep deprivation (SDep)**		
Genes	Correlation with time	Affected by SDep (vs. SS)
Tsc22d3, Zbtb16, Fkbp5, Lifr, Irs2, Ankrd50, Dnajb5, Hr, Reps2, Srxn1, Mfsd2a, Itga3, Pdia6, Setd7, Mc4r, Amigo2, Dpp9, Gtf2i, Uba6, Wnt2b, Sfpq, Zbtb40, Calr, 0610009O20Rik	positive	**↑ up-regulated with SDep**
Dbp	positive	**↓ down-regulated with SDep**
Nkx6–2, Slc6a6, Ddrgk1, Sox9, Rbm3, 2310022B05Rik, BC067074, Nckap5, Cirbp, Bzrap1, Naaa, Heyl	negative	**↓ down-regulated with SDep**

When expanding the correlation tests to all detected transcripts, 3 genes (*Nr1d2*, *Dbp*, and *Tef*) showed significant association with time during SS and 17 genes (including *Klf9*, *Tsc22d3*, and *Zbtb16*; see Additional file 3 for full list) showed significant association with time during SDep at an FDR cutoff of 5%. Although different individual genes were identified as being significantly correlated with time during SS and SDep, gene set enrichment analyses using the ranking of the correlation coefficients of all genes against the MSigDB database revealed positive enrichment of the pathways related to the circadian system and the circadian regulation of transcription for temporal changes during both SS and SDep (Additional file 3).

### Comparison to the genes differentially regulated between sleep and sleep deprivation in bulk tissue from mPFC

To gain further understanding of the sleep and wake regulation in VLPO-Gal neurons and explore differences from other brain regions, we utilized results from data collected from mPFC in a previous RNA-seq study using the same experimental design (i.e. SS vs. SDep over four time-points) [20]. Despite a number of dissimilarities between the two datasets, including brain regions (VLPO vs. mPFC), cell types (enriched galanin neuron vs. bulk tissue), and tissue preparation (micro-punch vs. LCM), 13,068 genes were detected in both studies, corresponding to 85% of the total detected genes. As expected, *Gal* and *galanin receptor 1* (*Galr1*) are among the 788 genes uniquely expressed in VLPO-Gal neurons, and cholinergic receptors (*Chrna1–5*) and microglia-specific genes involved in immune systems, such as C-type lectin family members (*Clec5a* and *Clec4a2*), are among the 1588 genes uniquely expressed in mPFC samples.

**Fig. 4:**
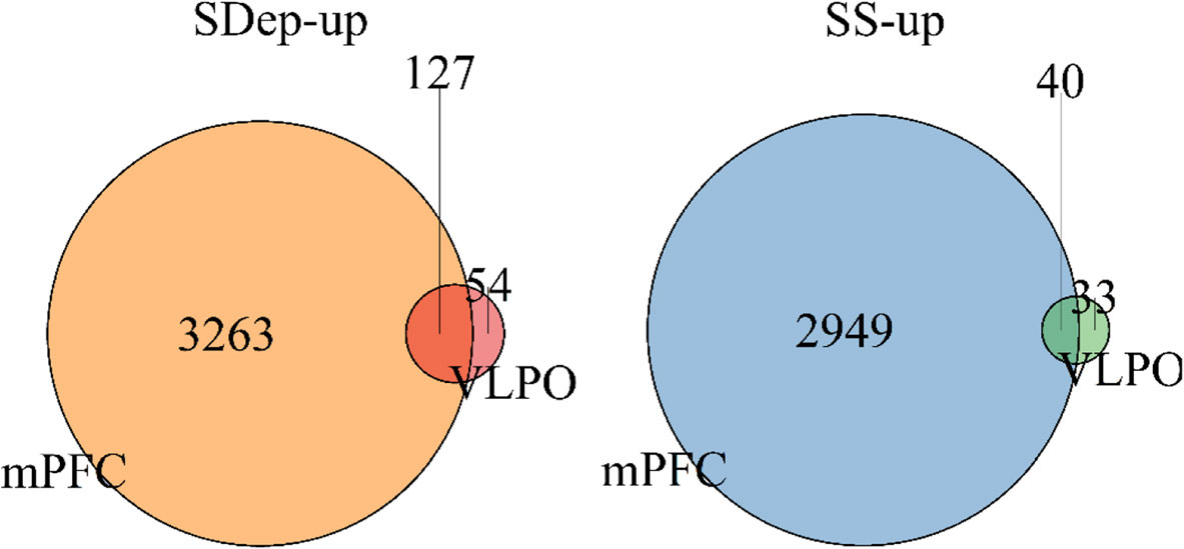
Venn Diagrams comparing DEGs identified in VLPO-Gal neurons and mPFC. The Venn diagram on the left shows 127 genes are commonly up-regulated by SDep in both VLPO-Gal neurons and mPFC. The Venn diagram on the right shows 40 genes are commonly up-regulated by SS in both VLPO-Gal neurons and mPFC. Specifically in VLPO-Gal neurons, 54 genes are up-regulated by SDep (left) and 33 genes are up-regulated by SS (right)

**Fig. 5:**
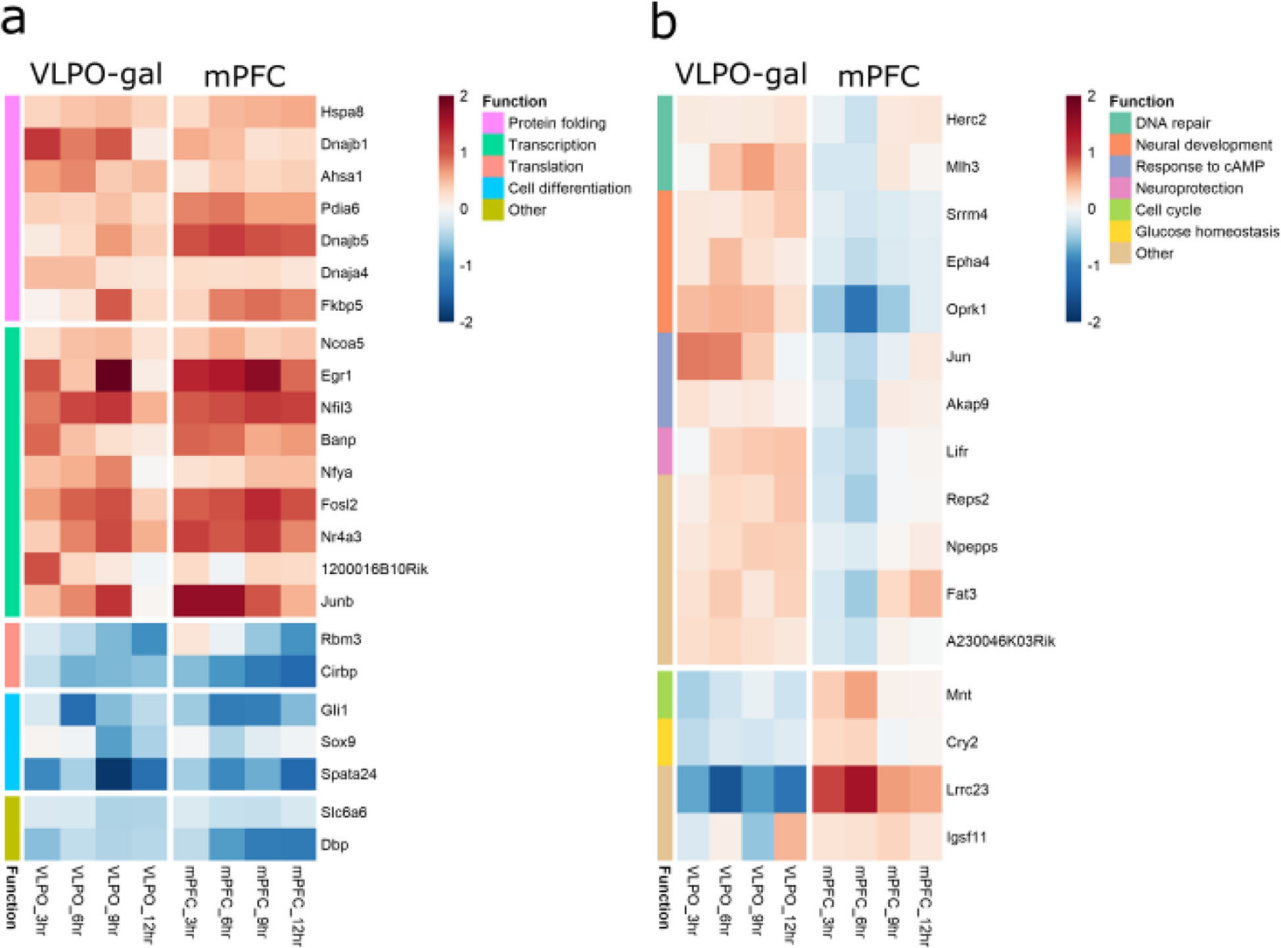
Heat map of fold-changes between sleep deprivation and sleep at each time point in VLPO-Gal neurons and mPFC. Cell colors with red indicates up-regulation with SDep and blue indicates up-regulation with sleep. Row annotations indicate biological functions. **a** Genes involved in protein folding and transcription are commonly up-regulated with SDep in both VLPO-Gal and mPFC, whereas translation and cell differentiation are commonly up-regulated with sleep in both VLPO-Gal and mPFC. **b** Sixteen genes show opposite direction of change between VLPO-Gal and mPFC cells. Twelve genes were up-regulated with SDep in VLPO-Gal neurons but up-regulated with sleep in mPFC. Functions played by these genes include DNA repair and neuronal development. Four genes (*Mnt*, *Cry2*, *Lrrc23*, and *Igsf11*) were up-regulated with sleep in VLPO-Gal but up-regulated with SDep in mPFC

Among the 13,068 genes commonly detected between the two studies, 254 genes (181 genes up-regulated by SDep and 73 genes up-regulated by SS) were significantly differentially regulated between sleep and SDep in VLPO-Gal neurons. Surprisingly, large proportions of these genes were also differentially regulated by SS or SDep in the same direction in mPFC, a region that has increased neuronal activity during wake. Specifically, 127 (70.0%) genes were commonly up-regulated by SDep in both VLPO-Gal and mPFC and 40 (54.8%) genes were found commonly up-regulated by sleep in both VLPO-Gal and mPFC (Fig. 4 and Additional file 4). As depicted in the heatmap of the fold-changes between SDep and SS across the four time-points from both studies (Fig. 5a), genes strongly up-regulated by SDep in both VLPO-Gal neurons and mPFC are involved in protein folding/ER stress and transcription regulation, including multiple heat shock genes (*Hspa8* and *Dnajb1*) and transcription factors (*Egr1* and *Fosl2*). Genes up-regulated by SS in both VLPO-Gal neurons and mPFC are involved in regulation of translation (cold-inducible RNA-binding proteins *Rbm3* and *Cirbp*), cell differentiation (*Gli1*, *Sox9*, and *Spata24*), beta-alanine transport (*Slc6a6*), and circadian rhythm (*Dbp*).

On the other hand, a subset of genes were regulated in opposite directions by SS and SDep between VLPO-Gal neurons and mPFC. Among the 54 genes up-regulated by SDep in VLPO-Gal neurons only, twelve were activated during SS in mPFC (Fig. 5b). These genes are involved in DNA repair (*Herc2* and *Mlh3*), nervous system development (*Epha4*, *Oprk1*, and *Srrm4*), cellular response to cAMP (*Akap9* and *Jun*), and neuroprotective signaling (*Lifr*). Similarly, among the 33 genes up-regulated by SS only in VLPO-Gal neurons, four genes (*Mnt*, *Cry2*, *Lrrc23*, and *Igsf11*) were activated by SDep in mPFC (Fig. 5b). *Max-binding protein* (*Mnt*) is involved in regulation of transcription and cell cycle, and *cryptochrome 2* (*Cry2*) is critical to glucose homeostasis and other functions related to circadian clock. Therefore, multiple mechanisms exist controlling transcription between SS and SDep in VLPO-Gal neurons. Some of these mechanisms may be systemic (e.g., due to neurohomornal effects or secondary to temperature change), while others could be region specific.

**Fig. 6:**
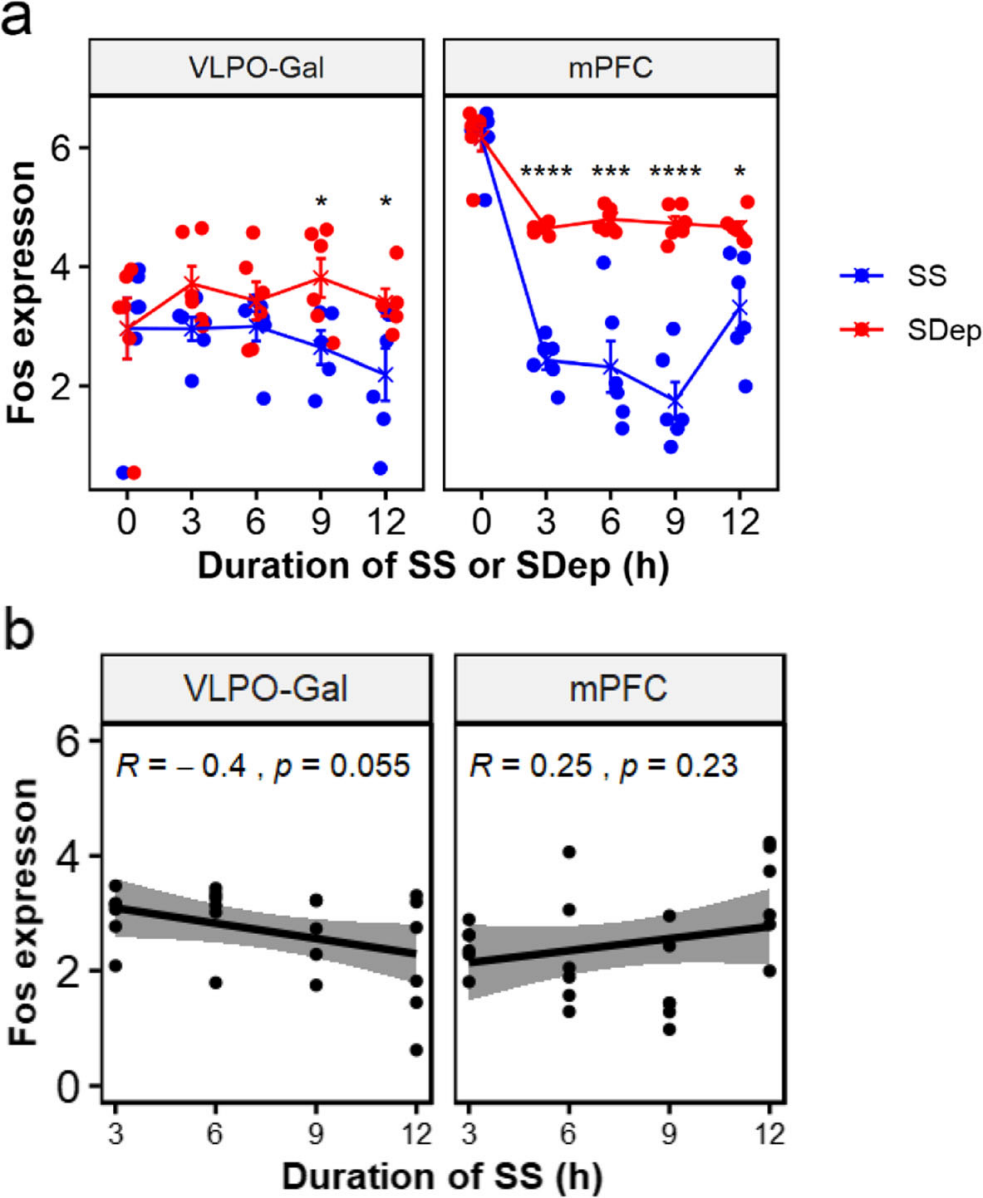
Expression of *Fos* across multiple time points during SS or SDep in VLPO-Gal neurons and mPFC. **a** Expression differences of *Fos* between SS and SDep. In VLPO-Gal, *Fos* was not significantly different between SS and SDep at earlier time points ZT3 and ZT6, but expressed at a higher level during SDep at ZT9 and ZT12 when compared using a two sample T-test. In mPFC, *Fos* was significantly elevated by SDep at all four time points. **b** Pearson’s correlation of *Fos* expression [log2(CPM + 0.5)] with duration of SS in VLPO-Gal (left) or mPFC (right). In VLPO-Gal, *Fos* showed a moderately large negative correlation with duration of SS (*R* = -0.40, *p* = 0.055), although not statistically significant. In mPFC, *Fos* was not significantly correlated with duration of SS but the direction of change was opposite from that of VLPO-Gal (*R* = 0.25, *p* = 0.23)

**Fig. 7:**
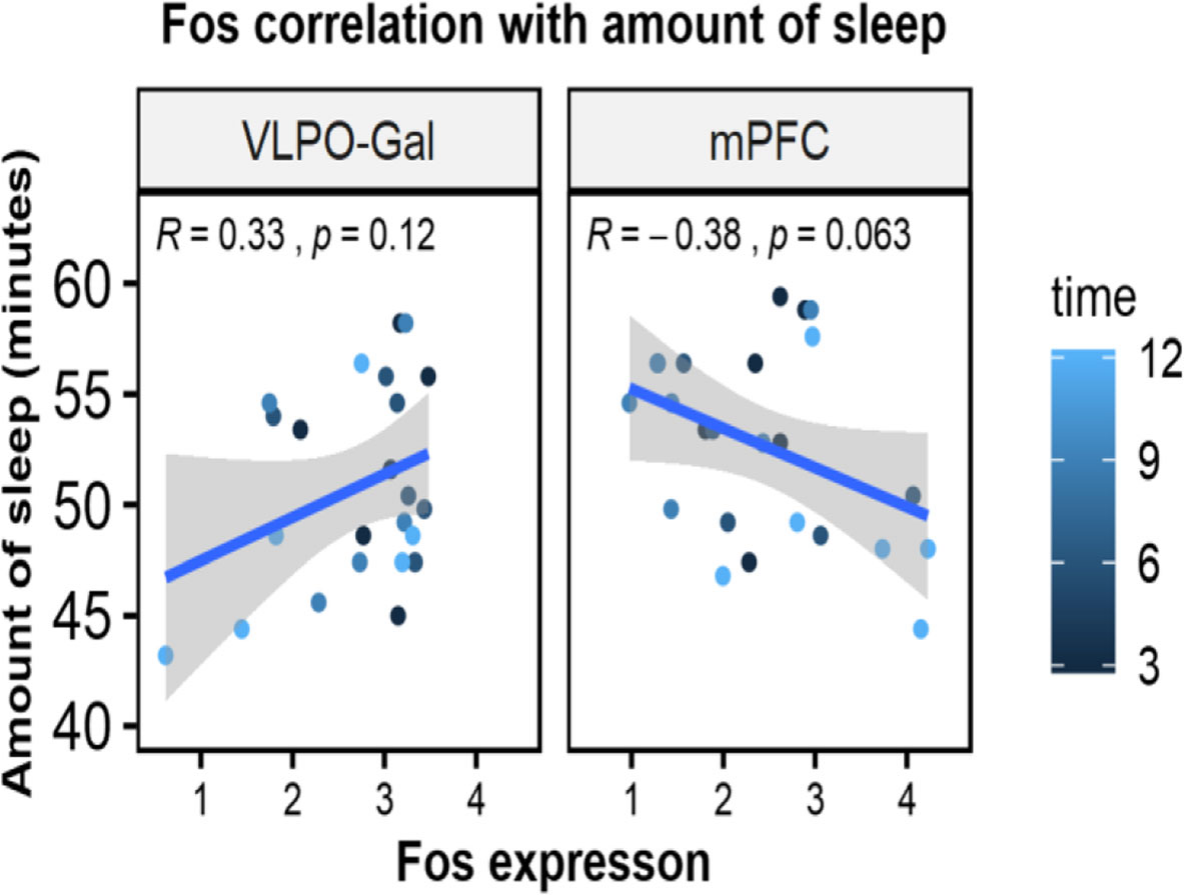
Pearson’s correlation of *Fos* with the amount (minutes) of sleep in the last 1 h before sacrifice in VLPO-Gal neurons and mPFC. *Fos* expression [log2(CPM + 0.5)] in VLPO-Gal had a moderate positive correlation (*R* = 0.33, *p* = 0.12) with the amount of sleep in the last hour before sacrifice, although not statistically significant. *Fos* expression in mPFC showed opposite direction of change as that of VLPO-Gal (*R* = -0.38, *p* = 0.063)

We next examined the *Fos* expression in VLPO-Gal neurons and mPFC. In VLPO-Gal neurons, *Fos* was not significantly differential between SS and SDep when compared across the four time points (FDR = 0.19). Therefore we compared the *Fos* levels at individual time-point between SS and SDep. *Fos* was expressed at a higher level with SDep with nominal significance when compared to the time-matched SS controls at later time points, ZT9 (*p* = 0.025) and ZT12 (*p* = 0.042) (Fig. 6a). The difference observed at these time points was driven by decreased *Fos* expression with increasing duration of SS toward the end of the light-phase (Pearson’s correlation coefficient with number of hours into sleep = − 0.40, *p* = 0.055; Fig. 6b), when lower sleep pressure is expected [12]. In contrast, in mPFC, expression of *Fos* was highly up-regulated by SDep across the four time-points (FDR = 4.94 × 10^− 20^) as well as at all four individual time points (Fig. 6a). *Fos* showed a trend of increase with duration of SS toward the end of the light-phase in mPFC (Pearson’s correlation coefficient = 0.25, *p* = 0.23), indicating that the trend of *Fos* expression decrease with duration of sleep is specific to the VLPO-Gal neurons. We then examined the correlation between *Fos* expression and the amount of sleep in the last hour before sacrifice in VLPO-Gal neurons and mPFC. We observed a trend of positive correlation between the *Fos* expression in VLPO-Gal neurons and amount of sleep (minutes) in the last hour before sacrifice (Pearson’s correlation coefficient = 0.33, *p* = 0.120; Fig. 7). While not statistically significant in our small sample, the observed correlation coefficient is moderately large, based on guidelines provided by Cohen for small (rho = 0.1), moderate (rho = 0.3) and large (rho = 0.5) correlations [11]. Thus, results suggest a moderate association between increased levels of *Fos* and greater sleep amounts in VLPO-Gal neurons. This correlation was found despite the limited range in sleep amounts among mice in our SS groups (72–97%; Fig. 7), which were required to have ≥70% in the last hour prior to sacrifice (see Methods). In contrast, in mPFC, *Fos* showed a trend of negative correlation with moderate association with the amount of sleep in the last hour before sacrifice (Pearson’s correlation coefficient = − 0.38, *p* = 0.063; Fig. 7). Taken together, these observations suggest that *Fos* shows region/cell-type specific trends in VLPO-Gal neurons with positive association with the sleep amounts before sacrifice and negative association with prolonged sleep or sleep need.

### VLPO-gal samples show significant enrichment of gene sets expressed in galanin-enriched inhibitory neurons clusters identified in a single-cell RNA-seq study

Although there is considerable evidence indicating that galanin cells in VLPO are sleep-active and sleep-promoting [17, 28, 49, 50], a recent study suggests that there might be a mixture of sleep-active and wake-active galanin neurons in VLPO [9]. To assess cellular heterogeneity, we utilized data from a recent single-cell RNA-seq study performed in the preoptic area of hypothalamus [38]. A number of galanin-enriched inhibitory (*n* = 5) and excitatory (*n* = 3) neuron clusters were identified. Among them, three inhibitory clusters (i8, i16, i18) and one excitatory cluster (e3) were localized at or near VLPO using multiplexed error-robust FISH (MERFISH). Genes highly expressed in these clusters were selected as gene sets (see Additional file 5 and Methods) and tested for enrichment in our LCM isolated VLPO-Gal samples. Two other inhibitory cell clusters were also considered: i2 [possibly containing the Tac1/Pdyn expressing sleep-active neurons identified by Chung et al. [9]] and i5 (enriched with Pou3f3 instead of galanin near VLPO). The gene sets informed by the galanin-expressing inhibitory neuron clusters i8 and i18 were the most significantly enriched in our VLPO-Gal samples (q = 1.9 × 10^− 5^ and 7.9 × 10^− 5^, respectively; Table 2), whereas gene sets based on the galanin-expressing excitatory neuron cluster e3 and the Tac1/Pdyn-expressing inhibitory neuron cluster i2 were not enriched in our VLPO-Gal samples (q = 0.15 and 0.052) (Table 2). As a sensitivity analysis to ensure that the enrichment results were not dependent on the specific gene set selection criteria or only limited to the chosen cell clusters, we tested enrichment of all 66 cell clusters identified in the single-cell RNA-seq dataset with varying criteria for gene selection (see Methods). The galanin-expressing inhibitory neuron cluster i8 was the most enriched cell cluster on average across sensitivity analyses, and the cell cluster i18 was mostly within the top 5 most enriched cell clusters and consistently more enriched than all other excitatory cell clusters or i2 and i5 (Additional file 5). These results support that our LCM isolated samples were enriched with galanin-expressing inhibitory neurons at or near VLPO, but not with galanin-expressing excitatory neurons or Tac1/Pdyn expressing inhibitory neurons primarily found outside of VLPO. These results, together with the positive association of *Fos* expression with the amount of sleep, suggest that our VLPO-Gal samples were primarily made up of sleep-active inhibitory neurons.

**Table 2:** Gene set enrichment analysis of gal-expressing neuron clusters identified in a single-cell RNA-seq study of preoptic area of hypothalamus [38]

Neuron Cluster	Description	Number of Genes	***p***-value	FDR
i8	Gal-inhibitory	25	3.17 × 10^−6^	1.90 × 10^− 5^
i18	Gal-inhibitory	27	2.64 × 10^− 4^	7.92 × 10^− 4^
i5	Pou3f3-inhibitory	39	1.75 × 10^−3^	3.50 × 10^− 3^
i16	Gal-inhibitory	31	7.78 × 10^−3^	1.17 × 10^−2^
i2	Tac1/Pdyn-inhibitory	54	4.38 × 10^−2^	5.25 × 10^− 2^
e3	Gal-excitatory	30	1.52 × 10^−1^	1.52 × 10^− 1^

Neuron cluster gene sets were adapted from [38] using criteria as described in Methods. Description indicates the marker gene enriched in the cluster and if the neuron cluster is inhibitory (starts with “i”) or excitatory (starts with “e”) (for detailed identities of the genes, see Additional file 5). *P*-value and FDR values are two-tailed *p*-value and Benjamini–Hochberg controlled FDR values obtained from the camera results, respectively

## Discussion

A total of 263 genes were differentially expressed between SS and SDep in VLPO-Gal neurons. Pathways related to protein folding/ER stress and regulation of transcription were enriched among the 184 genes up-regulated during SDep, whereas nucleosome assembly and regulation of translation were functions enriched among the 79 genes up-regulated during SS. A recent single-cell RNA-seq study of the preoptic area identified a number of galanin-enriched neuron clusters situated at or near VLPO [38]. Our gene set enrichment analysis demonstrated that our VLPO-Gal samples were highly enriched with genes expressed in inhibitory neuron cluster i8 (close to the core of VLPO), but not enriched with excitatory neuron cluster e3 (overlap with VLPO) nor the Tac1/Pdyn expressing inhibitory neuron cluster i2 [outside of VLPO, which is possibly the sleep-active neurons identified by Chung et al. [9]]. These results indicate that our LCM samples were enriched with galanin-expressing inhibitory neurons, but not the galanin-expressing excitatory neurons in VLPO. Earlier studies based on electrophysiological recording and c-Fos immunochemistry have shown that a majority of the sleep active neurons in VLPO are GABAergic and that the number of c-Fos positive GABAergic neurons correlates positively with the amount of sleep [16, 17]. In the current study, although not statistically significant, *Fos* expression showed a moderately large positive correlation [11] with the amount of sleep the animals experienced during the 1 h before sacrifice. These complimentary analyses indicate that our VLPO-Gal samples contained primarily sleep-active inhibitory neurons.

Genes involved in protein folding and ER stress pathways (*Hspa8*, *Hsph1*, and *Hspa5*) were significantly upregulated by SDep in VLPO-Gal neurons. Previous studies from us and others have demonstrated that these same genes/pathways are consistently up-regulated by wakefulness across multiple brain regions [cortex & hypothalamus [34], hippocampus [57], mPFC [20], and in wake-active Chat neurons of basal forebrain [42]], as well as in multiple peripheral tissues [liver [36], lung & heart [2], and pancreas [41]]. This confirms our previous hypothesis that a systemic mechanism is involved in the activation of protein-folding genes in response to extended wakefulness [42]. The activation of protein-folding genes is not specific to SDep or related to glucocorticoid signaling. Results from Cirelli et al. demonstrated the same heat-shock genes were also elevated during spontaneous wakefulness [10]. Similarly, heat shock genes were upregulated in sleep deprived adrenalectomized mice in which glucocorticoid signaling was maintained constant [39]. One possible mechanism for this molecular response is suggested by studies in *C. elegans* [55] that examined the effect of increasing expression of the spliced form of XBP-1 (XBP-1 s) in neurons. XBP-1 is one component of the unfolded protein response (UPR). XBP-1 s is a transcription factor that regulates expression of a number of genes involved in ER proteostasis [1, 48]. Transgenic expression of XBP-1 s in neurons in worms results in a cell-non-autonomous response in which the UPR is activated in multiple tissues [55]. While the basis of this effect was not fully identified, the mechanism involved neurotransmitter release from small vesicles, since the cell-non-autonomous UPR induction was attenuated in worms lacking UNC-13, which have a deficiency of release from small neuronal vesicles. The investigators did not, however, identify the actual signaling molecule [55].

Another set of genes that showed highly consistent changes between VLPO-Gal neurons and other brain regions were cold-inducible RNA-binding proteins (*Cirbp* and *Rbm3*). These genes were also elevated during sleep in peripheral tissues [2]. It has been speculated that expressions of these genes are controlled by the small drop in body temperature during sleep [42]. The change in body temperature across the day in mouse is largely caused by differences in behavioral state (sleep/wake) [21]. Another possible explanation of the systemic regulation of *Cirbp* across multiple brain regions and cell-types is its important function in circadian gene expressions [40]. A recent study demonstrated that *Cirbp* altered the changes in clock-gene expression produced by SDep [21]. Moreover, the amount of REM sleep during recovery following SDep is attenuated in *Cirbp* knockout mice. *Cirbp* is at the core of the interaction between circadian gene expression and sleep homeostasis.

Interestingly, a subset of genes were regulated between sleep and wake in the opposite direction in VLPO-Gal when compared to our previously published RNA-seq analysis of mPFC [20]. These genes included two involved in DNA repair, *Herc2* and *Mlh3*, which were elevated during SDep in VLPO-Gal samples, but activated during sleep in mPFC. This suggests that the expression of these genes is increased during relative quiescence of the relevant neurons (e.g., during sleep in mPFC and wake in galanin cells in VLPO). *Herc2* is an important mediator of DNA damage response and is critical for the ubiquitin-dependent retention of the DNA repair factors on damaged chromosomes [3]. *Mlh3* is important for DNA mismatch repairs [33]. Both genes have been implicated in cancer and neurodegenerative diseases [6, 26, 43, 63], indicating their essential roles in maintaining DNA integrity. Recent studies have established direct relationships between neuronal activity and DNA breaks, and DNA breaks in-turn facilitate the induction of immediate early genes [35]. Another study used time-lapse imaging of chromosomal markers of live zebrafish and revealed accumulation of DNA breaks during wakefulness and increased chromosome dynamics (essential for DNA damage repair) during sleep [61]. This is consistent with our finding of elevated DNA repair pathways during sleep in mPFC. In contrast, VLPO-Gal neurons show increased activity during sleep, and reduced activity during wakefulness [17, 50]. This is likely the reason why we observed increased DNA repair during wakefulness in VLPO-Gal neurons.

By comparing the genes differentially regulated between sleep and wake in mPFC to those in VLPO-Gal neurons, there was a large reduction in the extent of transcriptional regulation in VLPO-Gal cells. Among the 13,068 genes commonly detected between the two studies, 6179 (47.3%) had significantly altered expressions between SS and SDep in mPFC, whereas only 254 genes (1.9%) were differentially regulated in VLPO-Gal cells at the same FDR cutoff of 5%. Similarly, in our previous microarray studies, 3988 and 823 genes were identified to be differentially regulated between sleep and wake in cortex and hypothalamus bulk tissues, respectively [34]. A previous study of Chat neurons in basal forebrain also revealed a small number of genes (*n* = 10) meeting an FDR cutoff of 5% [42]. These results are consistent with the findings from an in-situ hybridization whole-brain mapping of the effect of SDep [56], which demonstrated that hypothalamus and brainstem are relatively less responsive at the transcriptional level compared to cortex and hippocampus.

Our study is the first to investigate the transcriptional changes between sleep and wake in galanin neurons from VLPO, a region/cell-type that plays important roles in sleep regulation. Although we cannot completely eliminate the possibility of cross-contamination from other cell-types, such as glia cells, our LCM isolated samples showed > 100-fold enrichment of galanin and > 70-fold depletion of *Aldh1l1*, a marker of the astrocyte. Therefore, we are confident to conclude that the changes observed are primarily coming from the galanin-expressing neurons. However, there are several limitations that merit discussion. A technical limitation is the use of LCM to isolate GFP-labeled cells requiring fixation using formaldehyde, which has the potential to cause RNA damage [19]. Since the effect of formaldehyde on RNA integrity is temperature and time dependent [13, 31, 58], we performed post-fix on ice for only 5 min, which minimizes the influence of formaldehyde. For example, one study has shown that the quality of RNA derived from white blood cells incubation with formaldehyde is stable for as long as 3 days at 4 °C [31]. In addition, we used an optimized RNA extraction buffer for fixed tissues that contains proteinase K and SDS and obtained good quality RNA samples (mean RIN value of 8.21). However, despite these approaches, formaldehyde may still cause RNA modifications and variations in the captured RNA reads [24]. Therefore, in comparisons between our current data and previously acquired RNA-seq data from fresh-frozen mPFC mouse tissues, rather than directly comparing the RNA reads, we restricted the comparisons to the differentially expressed genes identified from the two studies. Another technical challenge of the study is the heterogeneous nature of the hypothalamus. A single-cell RNA-seq study of hypothalamus identified 34 neuronal clusters [8]. Another single-cell RNA-seq study of the preoptic area of hypothalamus identified 43 inhibitory neuron clusters and 23 excitatory neuron clusters [38]. This highlights the need to study cell-type specific gene expression changes in highly heterogeneous regions; our LCM study serves as an initial approach in this direction. However, while more uniform than bulk tissue, a limitation in our data is that the results are still obtained from pooling of hundreds of cells. A future single-cell RNA-seq study is required to further decipher the transcriptomic changes responding to sleep and wake at the single cell level. Finally, a limitation of our study design is the lack of a condition that can dissociate homeostatic sleep pressure from wakefulness. *Fos* showed a trend of decreasing with longer time spent asleep toward the end of the light phase, a time where *Fos* was also expressed at higher levels under SDep compared to time-matched SS controls. These observations agree with the previous findings that the activities of VLPO-Gal neurons correlate to sleep need and increase under sleep pressure induced by SDep [17, 50]. However, since SDep increases both sleep pressure and wakefulness, the current study is not designed to specifically confirm this conclusion, as we cannot exclude the possibility that VLPO-Gal neurons are activated by wakefulness or by both sleep pressure and wakefulness. A future study should also examine expression changes of VLPO-Gal neurons under recovery sleep after SDep to investigate the transcriptional regulation involved in homeostatic sleep control.

## Conclusions

In conclusion, our results indicate that at the transcriptional level both wake-active and sleep-active neurons show increased expression of genes in the endoplasmic reticulum unfolded protein response during wakefulness, while during sleep they show increased expression of genes for cold-induced RNA binding proteins. Thus, these effects are systemic and indeed found in organs other than brain [2, 36, 41]. In contrast, we found that expression of DNA repair genes is increased in VLPO-Gal cells during wakefulness, but increased in wake-active cells during sleep [4, 20]. The increase in expression of these genes during the cell-type-specific quiescent periods suggests expression may be driven by region or cell-type specific activity – DNA breaks occur when neurons are active and are repaired when neurons are relatively inactive. The role of these changes in regulation of sleep and wake remains to be determined.

## Methods

### Mouse experiments

Male Tg (Gal-EGFP)HX109Gsat mice on a Swiss-Webster background that express eGFP under the control of galanin promoter were obtained from the Mutant Mouse Resource & Research Centers. Mice at 2–3 months of age were housed individually in a pathogen-free, temperature (22 °C), and humidity (45–55%) controlled room with a 12-h/12-h light/dark cycle with lights-on at 7 AM (Zeitgeber time zero or ZT0). Water and food were available ad libitum. Animals were acclimated for 14 days to individual housing and the presence of experimenter prior to the experiment. There were two groups of mice—one that was sleep deprived by gentle handling and one that was allowed to sleep spontaneously. On the days of the experiments, SDep was initiated using gentle handling [14] at lights-on, and mice were collected following 3, 6, 9, and 12 h of SDep (*n* = 5–6 at each time point). For mice that were allowed SS at matching time-points (*n* = 5–6 at each time point), sleep was monitored starting from lights-on; only mice that slept for ≥70% of the last 1 h before sacrifice were included in the SS group. Sleep was monitored using the AccuScan infrared monitoring system that detects movement when the mouse crosses electronic beams, and based on previous validation studies performed with Swiss-Webster male mice of 3 months of age using EEG/EMG, sleep was defined as ≥50 s of continuous inactivity [42]. Additional mice (*n* = 6) were also chosen randomly for “baseline” assessment of gene expression and sacrificed at ZT0. In total, 52 mice were used for the study of gene expression differences between SS and SDep. An additional seven animals under undisturbed conditions were collected during the light phase and were used in the study to test for the enrichment of galanin neurons in isolated eGFP(+) cells. Therefore, a total of 59 mice were used in this study.

### Tissue collection

Mice were euthanized by cervical dislocation and brains were quickly removed and rinsed in nuclease-free 1x phosphate buffered saline (PBS). A 3 mm coronal section (Bregma + 1.5 to − 1.5) containing the ventrolateral preoptic (VLPO) region was incubated in ice-cold 4% formaldehyde (pH 7.4) for 5 min, rinsed twice with PBS, frozen in cryo-embedding medium OCT (Tissue-Tek), and stored at − 80 °C until sectioning. 10 μm sections were made in a cryostat kept at − 20 °C. The VLPO region was identified based on the mouse atlas of Paxinos and Franklin’s [15]. Using the anterior commissure as a landmark and the eGFP labeled galanin neurons as a guidance, approximately 50 brain slices containing the VLPO region (Bregma + 0.40 to − 0.10) per animal were captured on regular glass microscope slides, 2–3 sections per slide, and stored in covered slide box and sealed in plastic bags at − 80 °C until use of LCM.

### Laser capture microdissection (LCM)

LCM was performed as described previously [42]. Briefly, brain sections were dehydrated immediately before using the LCM protocol: air dry (30 s), 75% ethanol (30 s), RNase-free water (15 s), 75% EtOH (30 s), 95% EtOH (30 s), 1st 100% ethanol (30 s), 2nd 100% ethanol (1 min), 1st xylene (1 min), and 2nd xylene (3 min), and finally air dried in hood for 5 min to remove residual xylene. Between each transition from buffer to buffer, the slides were quickly tapped against the tube rim to remove excess liquid. Dehydrated slides were kept in clean and dried microscope slide boxes containing molecular sieves until ready for LCM. Gal-eGFP(+) neurons from VLPO were dissected using Arcturus XT LCM system (MDS analytical technologies). Since the short 5 min post-fix did not result in deep penetration of formaldehyde, neurons in the dorsal extension of VLPO and the medial and median preoptic nuclei were mostly not visible, whereas GFP fluorescent at the VLPO core and the medial extension were well preserved. GFP-labeled cells were dissected primarily from the VLPO core with care taken to avoid picking galanin neurons from the VLPO medial extension based on the staining of all galanin neurons in POA described elsewhere [28]. The thermoplastic film on the cap was carefully peeled off and immersed in 100 μl lysis buffer (10 mM Tris-HCl [pH 7.5], 50 mM EDTA [pH 8.0], 0.2 M NaCl, 2.2% SDS, RNase inhibitor and 1000 mg/ml proteinase K) and incubated at 55 °C for 30 min with gentle shaking. This lysis method was adapted based on an optimized method for fixed tissues [25]. After lysis, the tube was stored at − 80 °C until RNA extraction.

### RNA purification, sequencing and bioinformatics

RNA extraction was performed using an RNAqueous-Micro Total RNA Isolation Kit (Invitrogen) and on-column DNase digestion was performed using an RNase-Free DNase Set (Qiagen) following the manufacturers’ protocols. RNA concentration and qualities were accessed by Agilent 2100 Bioanalyzer RNA 6000 Pico chip. All samples had RIN values above 7.0, with a mean (standard deviation) RIN of 8.21 (0.52). Library preparation was performed using a SMARTer® Stranded Total RNA-Seq Kit v2 - Pico Input Mammalian by Takara Bio, and sequencing done with 150 base-pair paired-end reads on Illumina HiSeq 4000. Raw reads were aligned to the mouse genome build mm9 by STAR version 2.5.3a, and quantified at the gene level using scripts from the PORT pipeline (github.com/itmat/Normalization -v0.8.4-beta). Low-expressing genes were removed by keeping the genes with mean of 10 counts or higher across all samples. A total of 13,856 genes with unique ensemble IDs that passed this filtering criteria were normalized using the “Trimmed Mean of M-values” (TMM) method in the *edgeR* package [45], and differential expression analyses were performed using the *LimmaVoom* package [44] (see also *Differential gene expression and functional analysis*, below).

### Test enrichment of galanin in eGFP(+) samples

eGFP(+) neurons from VLPO core were collected using LCM from four animals collected during undisturbed conditions during light phase as described above following the same procedures. eGFP(−) cells were collected from the adjacent piriform cortex region on the same sections in the same animals using LCM. VLPO bulk tissue was collected from the remaining three mice, which were euthanized by cervical dislocation and whole brains were quickly removed and frozen on dry ice. VLPO region bulk tissue (Bregma + 0.4 to − 0.1, lateral ±0.5 mm) was punched using a 0.7 mm micropunch based on the mouse atlas of Paxinos and Franklin’s [15] in a cryostat and using the anterior commissure as a landmark. This micropunch method has previously been used for our studies of mPFC [20]. Enrichment of galanin expressing neurons and depletion of astrocytes in eGFP(+) samples were tested by comparing expression of *Gal* and *Aldh1l1* in eGFP(+) samples (*n* = 4), eGFP(−) samples (*n* = 4), and the VLPO bulk tissue (*n* = 3). Real time PCR was performed using the following TaqMan assays: *Gal* (Mm00439056_m1), *Aldh1l1* (mm03048957_m1), *Tbp* (mm00446971_m1), and *Hprt* (mm01545399_m1). Relative expressions of *Gal* and *Aldh1l1* were calculated using the 2-ΔΔCT method [47] with the reference values being the geometric means of the two housekeeping genes, *Tbp* and *Hprt*. Comparisons of *Gal* and *Aldh1l1* expression were made among the LCM samples [eGFP(+) or eGFP(−)] and the VLPO bulk samples on the –ΔCt level using an exact *p*-value from the Kruskal-Wallis test. To test for enrichment of *Gal* in eGFP(+) samples, two separate pairwise comparisons were made: eGFP(+) vs. eGFP(−) and eGFP(+) vs. VLPO bulk samples, using a one-tailed Wilcoxon exact test, with median and 95% confidence intervals of differences derived using the Hodges-Lehmann estimate. Similarly, pairwise comparisons of eGFP(+) vs. VLPO bulk and eGFP(−) vs. VLPO bulk were performed to test for depletion of *Adlh1l1* in LCM samples compared to the bulk tissue. Analyses were performed using R (www.r-project.org) and SAS Version 9.4 (SAS Institute, Cary, NC).

### Differential gene expression between sleep and sleep deprivation

Using the *LimmaVoom* package in R software [30], we evaluated the differentially expressed genes (DEGs) between sleep deprived and spontaneous sleep behavioral states (SDep vs. SS) across all time points. Specifically, SDep samples were compared with their time-matched SS controls at each of the four time durations (3, 6, 9, and 12 h), and a moderated F-test was used to assess a global statistical difference between SS and SDep for each gene combining t-statistics for all contrasts at individual time points [44]. Multiple comparisons correction for statistical significance was applied using the method of Benjamini and Hochberg [5] to control the overall false discovery rate (FDR) at 5%. A multidimensional scaling (MDS) plot of all samples was generated using the plotMDS function in Limma with default parameters. The Euclidean distance between each pair of samples, calculated as the root-mean-square deviation of the top 500 most variable genes automatically selected for each pair of samples, was used as an indication of the similarity between samples [44]. The first dimension represents the leading-fold-change that best separates samples and explains the largest proportion of variation in the data, with subsequent dimensions having a smaller effect and being orthogonal to the ones before it [30].

### Temporal expression changes during sleep and sleep deprivation

To explore the time-dependent changes in gene expression during sleep and sleep deprivation, Pearson’s correlation coefficients were calculated between ordinal time (ZT0, ZT3, ZT6, ZT9, and ZT12) and gene expression values, combining animals (*n* = 6) collected at lights-on as time zero and animals collected during SS and SDep across the four time points (ZT3, 6, 9, and 12), separately. Gene expression correlation with time was first tested for the DEGs differentially regulated between SS and SDep, with statistical significance based on an FDR correction following the method of Benjamini and Hochberg [5]. Secondly, as a broader discovery analysis gene expression correlation with time was tested for all detected genes. The correlation coefficients obtained were ranked from highest to lowest and pathway enrichment was explored using the gene set enrichment test via Camera [59] against the MSigDB gene sets for mouse (v5.2) [32].

### Comparison to RNA-seq data of medial prefrontal cortex (mPFC)

Data and results were obtained from a previously published RNA-seq study performed on mPFC using the same experimental design [20]. Differentially regulated genes between SS and SDep in mPFC identified from the young animals (2–4 months) were used to compare with data obtained from this study. The mPFC data are publicly available at Gene Expression Omnibus (GSE128770).

### Functional analysis

Functional gene ontology (GO) analysis was performed using DAVID Bioinformatics Resources 6.8 [22]. Unless otherwise noted, enrichment of GOs was performed against GOTERM_BP_Direct (Biological Process), GOTERM_MF_Direct (Molecular Function), and GOTERM_CC_Direct (Cellular Component). For DEGs identified in VLPO-Gal neurons, the GO enrichment tests were performed for the genes up-regulated by SDep and the genes up-regulated by SS, separately. For DEGs also differentially regulated in the same direction in mPFC, the GO enrichment tests were performed for the common up-regulated genes by SDep and the common up-regulated genes by SS, separately. Enrichment of GO terms was defined as a *p* < 0.05 and containing at least 2 genes. The 13,856 genes used for identifying DEGs were used as the background in all cases.

### Gene set enrichment analysis

Gene sets of different neuronal clusters were chosen based on the preoptic area single-cell RNA-seq data by Moffitt et al. [38]. The following neuron clusters were chosen: galanin-enriched inhibitory neurons at or near VLPO (i8, i16, i18), gal-enriched excitatory neurons near VLPO (e3), Tac1/Pdyn-enriched inhibitory neurons in horizontal limb of the diagonal band of Broca (HDB) (i2), and Pou3f3-enriched inhibitory neurons at VLPO (i5). The marker gene set of a given cell cluster was defined as having 10% higher expression than the mean expression across all the cell clusters and being detectable in > 25% of all cells in the corresponding cell cluster [7]. The resulted gene sets are shown in Additional file 5. Gene set enrichment was run using Camera from the *Limma* package using default parameters against a gene list ranked based on mean expression averaged across all VLPO-Gal samples [59]. The gene set enrichment analysis was further tested across 18 different selection criteria to explore if the enrichment results were stable. Selection criteria were chosen as combinations of three possible expression levels (5, 10%, or 20% higher than mean) and six possible detection levels (> 15, 20, 25, 30, 40%, or 50% of cells).

### Association of Fos expression with sleep

Previous studies relying on c-Fos immunoreactivity demonstrated increased c-Fos expression in VLPO with greater amounts of sleep [17, 50]. Here, we calculated Pearson’s correlations to test for an association between *Fos* expression and minutes of sleep in the last hour prior to sacrifice. This was done for the studies reported here and from our previous data on mPFC.

### Power calculation

Power calculation for the RNA-seq study was performed using the RnaSeqSampleSize package and its web interface (https://cqs-vumc.shinyapps.io/rnaseqsamplesizeweb/) [62]. Based on our prior data and results in mPFC [20], we expected to detect 14,000 genes, and that up to 5000 may be differentially expressed between conditions. At an FDR of 5%, a total of 48 animals (*n* = 24 per condition) would provide 84% power to detect a minimum fold-change of 2.0 between groups.

## Supplementary information

**Additional file 1: Figure S1**. *Distribution of galanin neurons in VLPO in post-fix mouse brain.* Galanin neurons have been shown to be distributed in a few different structures besides the VLPO core (VLPOc). These include the medial (VLPOem) and dorsal (VLPOed) extensions, as well as the median (MnPO), medial (MPO), and periventricular preoptic (PvPO) nuclei [28]. **A** shows a reference galanin neuron distribution at around Bregma + 0.14, for which the brain was prepared using perfusion with 10% formalin. **B** shows a brain slide at approximately the same location from a brain sample prepared using 5 min post-fix with 4% formaldehyde, the same as the samples used for LCM in this study. **C** shows the enlarged image containing the GFP-labeled galanin neurons. The different structures were drawn based on the reference image. The outlines were drawn in the absence of Nissl staining and were for illustration only. As shown in the images (**B** and **C**), the short post-fix allowed well penetration of formalin (fix the GFP protein) primarily in the VLPO-core but not deep enough to reach the dorsal extension (VLPOed), MPO, MnPO, or the dorsal part of the PvPO. AC, Anterior commissure; 3 V, 3rd ventricle; OC, Optic chiasm. **Figure S2**. *Identification and dissociation of eGFP-galanin neurons from VLPO core using LCM*. **A** shows a representative brain section at approximately + 0.15 Bregma under fluorescence with the different structures illustrated based on the reference image shown in Supplementary figure 1. The outlines were drawn in the absence of Nissl staining and were for illustration only. **B** shows the same brain slide after the eGFP (+)-galanin cells being removed in the VLPO core structure. Care was taken to avoid selecting other eGFP(+) cells in the surrounding structures and regions. The purple circles indicate the exact location where the cells were dissociated. The image is directly taken from the LCM instrument. **C** shows the captured cells on the CapSure HS cap.

**Additional file 2.**

**Additional file 3.**

**Additional file 4.**

**Additional file 5.**

## Data Availability

All raw and processed RNA-seq data generated in this study have been submitted to the NCBI Gene Expression Omnibus (GEO) under accession number GSE132308 (https://www.ncbi.nlm.nih.gov/geo/query/acc.cgi?acc=GSE132308). The mPFC data are publicly available at GEO (https://www.ncbi.nlm.nih.gov/geo/query/acc.cgi?acc= GSE128770) using the mouse (*Mus musculus*) genome build NCBI37/mm9 assembly by the Mouse Genome Sequencing Consortium (https://www.ncbi.nlm.nih.gov/assembly/GCF_000001635.18/).

## Abbreviations

BP: Biological process
CC: Cellular component
DEGs: Differentially expressed genes
DR: Dorsal raphe
EEG/EMG: Electroencephalogram/electromyography
eGFP: Enhanced green fluorescent protein
ER: Endoplasmic reticulum
FDR: False discovery rate
Gal: Galanin
GO: Gene ontology
HDB: Horizontal limb of the diagonal band of Broca
LC: Locus coreleus
LCM: Laser captured microdissection
MERFISH: Multiplexed error-robust fluorescence in situ hybridization
MF: Molecular function
mPFC: Medial prefrontal cortex
NREM: Non-rapid eye movement
POA: Preoptic area
REM: Rapid eye movement
RNA-seq: RNA-sequencing
SDep: Sleep deprivation
SS: Spontaneous sleep
TMN: Tuberomammillary nucleus
VLPO: Ventrolateral preoptic nucleus
