# Supplementary material for: RNA-seq analysis of galaninergic neurons from ventrolateral preoptic nucleus identifies expression changes between sleep and wake
Source: BMC Genomics. 2020 Sep 14;21:633. doi: 10.1186/s12864-020-07050-7 (PMC7491139; doi:10.1186/s12864-020-07050-7)
Supplement: Supplementary file 1 — Additional file 1: Figure S1. Distribution of galanin neurons in VLPO in post-fix mouse brain. Galanin neurons have been shown to be distributed in a few different structures besides the VLPO core (VLPOc). These include the medial (VLPOem) and dorsal (VLPOed) extensions, as well as the median (MnPO), medial (MPO), and periventricular preoptic (PvPO) nuclei [28]. A shows a reference galanin neuron distribution at around Bregma + 0.14, for which the brain was prepared using perfusion with 10% formalin. B shows a brain slide at approximately the same location from a brain sample prepared using 5 min post-fix with 4% formaldehyde, the same as the samples used for LCM in this study. C shows the enlarged image containing the GFP-labeled galanin neurons. The different structures were drawn based on the reference image. The outlines were drawn in the absence of Nissl staining and were for illustration only. As shown in the images (B and C), the short post-fix allowed well penetration of formalin (fix the GFP protein) primarily in the VLPO-core but not deep enough to reach the dorsal extension (VLPOed), MPO, MnPO, or the dorsal part of the PvPO. AC, Anterior commissure; 3 V, 3rd ventricle; OC, Optic chiasm. Figure S2. Identification and dissociation of eGFP-galanin neurons from VLPO core using LCM. A shows a representative brain section at approximately + 0.15 Bregma under fluorescence with the different structures illustrated based on the reference image shown in Supplementary figure 1. The outlines were drawn in the absence of Nissl staining and were for illustration only. B shows the same brain slide after the eGFP (+)-galanin cells being removed in the VLPO core structure. Care was taken to avoid selecting other eGFP(+) cells in the surrounding structures and regions. The purple circles indicate the exact location where the cells were dissociated. The image is directly taken from the LCM instrument. C shows the captured cells on the CapSure HS cap. [file 12864_2020_7050_MOESM1_ESM.docx]

Additional file 1


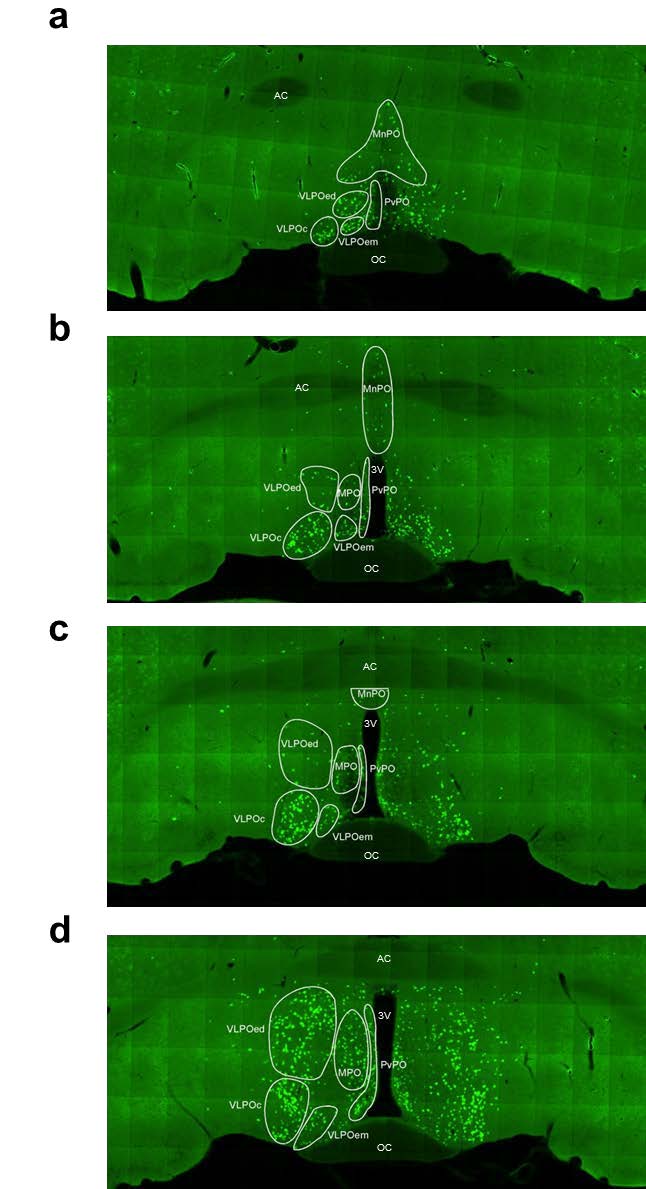

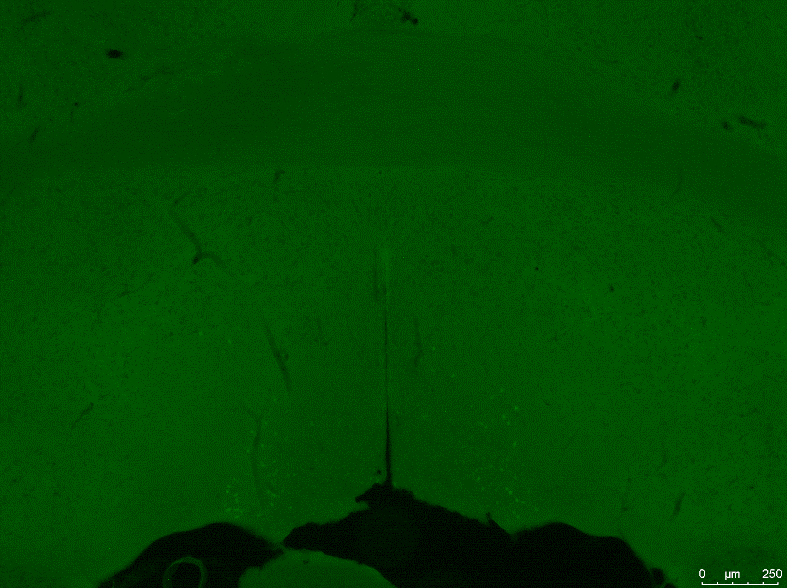


C

A

B

VLPOem

VLPOed

MPO

VLPOc

PvPO

AC


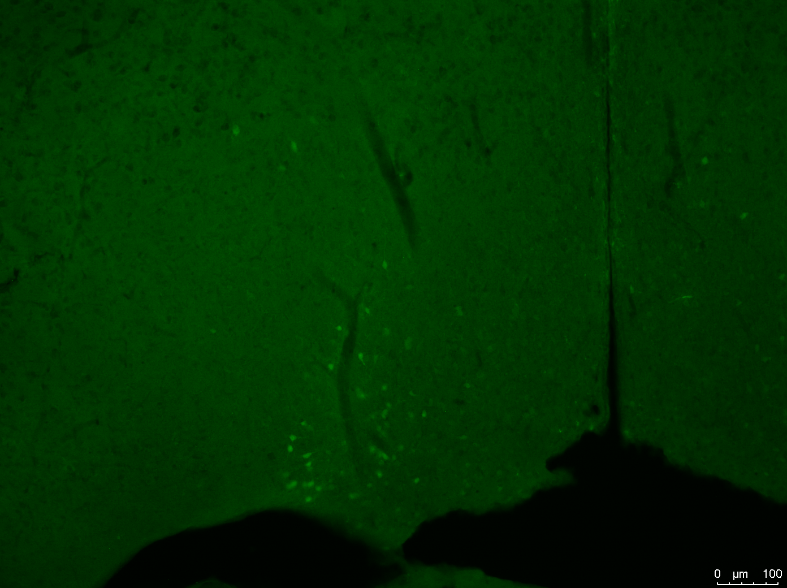


VLPOem

VLPOed

MPO

VLPOc

PvPO

**Supplementary figure 1**. *Distribution of galanin neurons in VLPO in post-fix mouse brain.* Galanin neurons have been shown to be distributed in a few different structures besides the VLPO core (VLPOc). These include the medial (VLPOem) and dorsal (VLPOed) extensions, as well as the median (MnPO), medial (MPO), and periventricular preoptic (PvPO) nuclei (Kroeger, Absi et al. 2018). **A** shows a reference galanin neuron distribution at around Bregma +0.14, for which the brain was prepared using perfusion with 10% formalin. **B** shows a brain slide at approximately the same location from a brain sample prepared using 5min post-fix with 4% formaldehyde, the same as the samples used for LCM in this study. **C** shows the enlarged image containing the GFP-labeled galanin neurons. The different structures were drawn based on the reference image. The outlines were drawn in the absence of Nissl staining and were for illustration only. As shown in the images (**B** and **C**), the short post-fix allowed well penetration of formalin (fix the GFP protein) primarily in the VLPO-core but not deep enough to reach the dorsal extension (VLPOed), MPO, MnPO, or the dorsal part of the PvPO. AC, Anterior commissure; 3V, 3rd ventricle; OC, Optic chiasm.


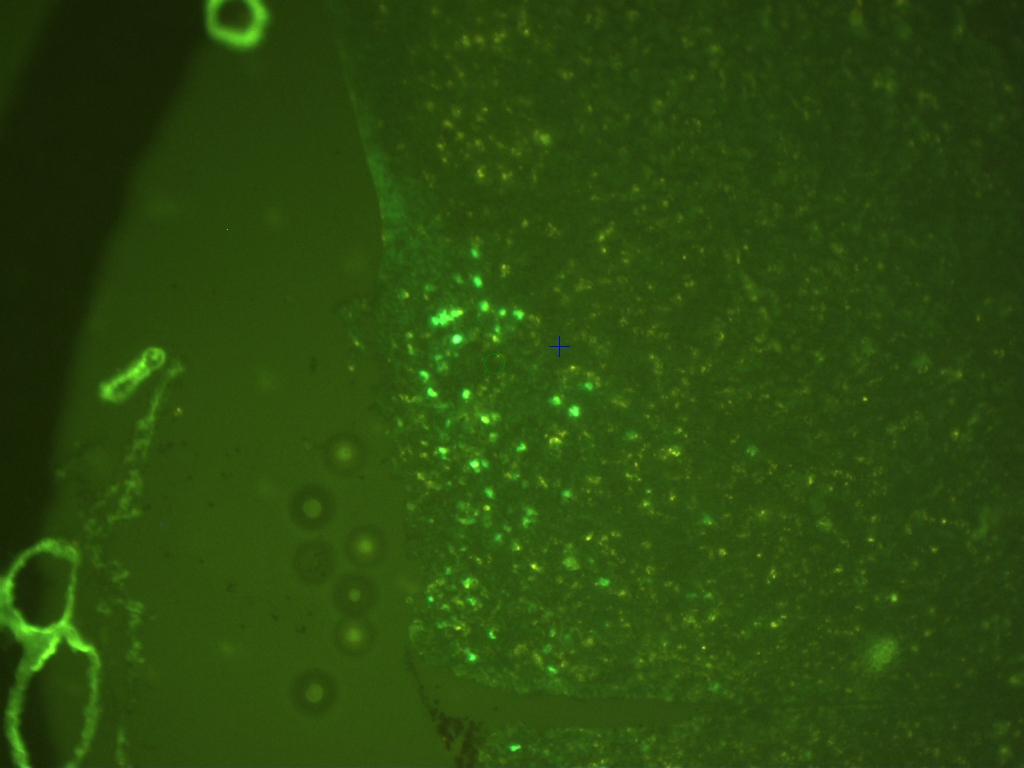

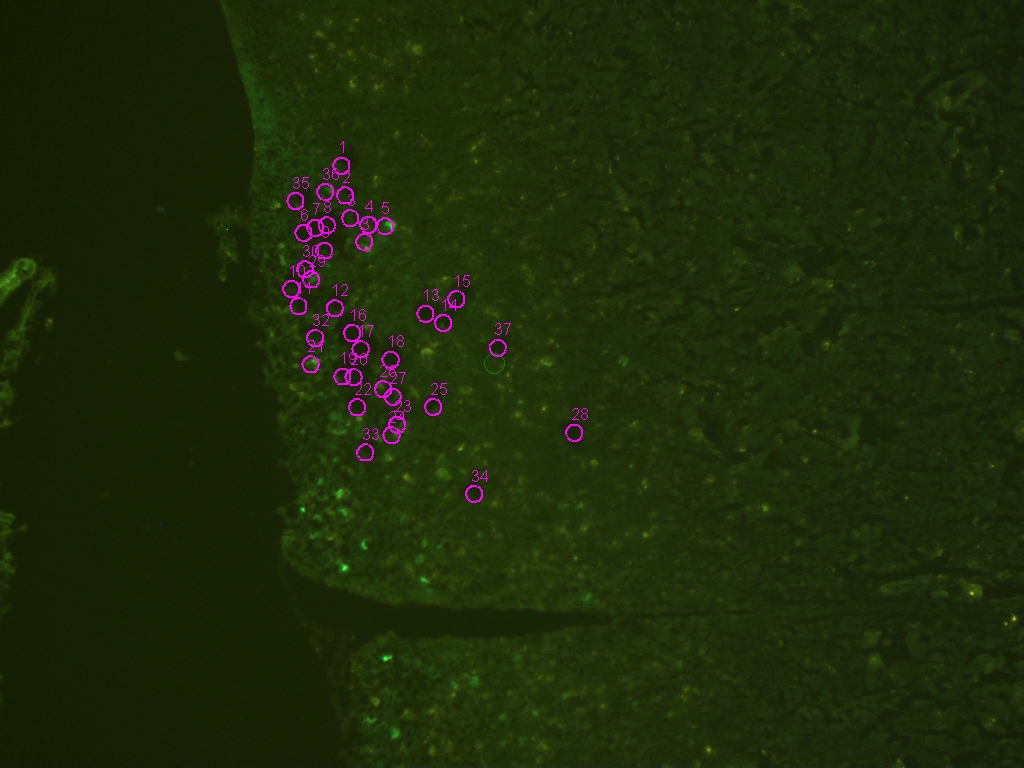

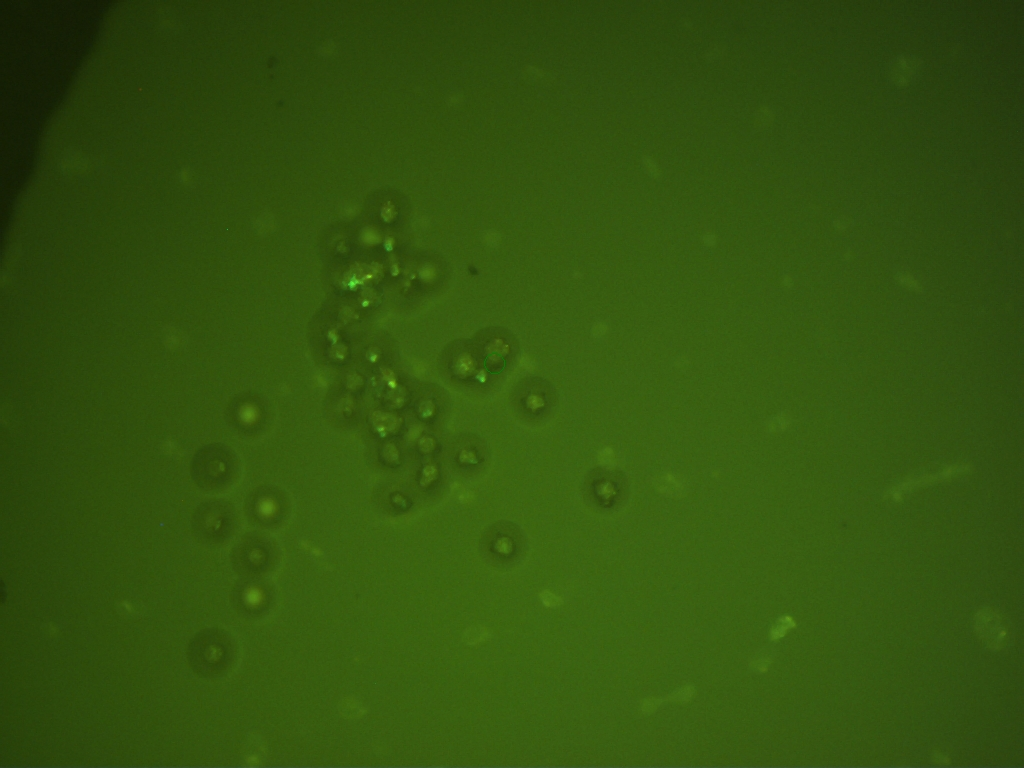


C

A

B

VLPOem

VLPOed

MPO

VLPOc

PvPO

VLPOem

VLPOed

MPO

VLPOc

PvPO

**Supplementary figure 2**. *Identification and dissociation of eGFP-galanin neurons from VLPO core using LCM*. **A** shows a representative brain section at approximately +0.15 Bregma under fluorescence with the different structures illustrated based on the reference image shown in Supplementary figure 1. The outlines were drawn in the absence of Nissl staining and were for illustration only. **B** shows the same brain slide after the eGFP (+)-galanin cells being removed in the VLPO core structure. Care was taken to avoid selecting other eGFP(+) cells in the surrounding structures and regions. The purple circles indicate the exact location where the cells were dissociated. The image is directly taken from the LCM instrument. **C** shows the captured cells on the CapSure HS cap.

Reference:

Kroeger, D., G. Absi, C. Gagliardi, S. S. Bandaru, J. C. Madara, L. L. Ferrari, E. Arrigoni, H. Munzberg, T. E. Scammell, C. B. Saper and R. Vetrivelan (2018). "Galanin neurons in the ventrolateral preoptic area promote sleep and heat loss in mice." Nat Commun **9**(1): 4129.
